# Sex Differences in COVID-19 Outcomes

**DOI:** 10.7759/cureus.25760

**Published:** 2022-06-08

**Authors:** Karim El Aidaoui, Rita Ait Benhamou, Amal Haoudar, Jihane Ziati, Aziza Kantri, Khalid Agrad, Chafik El Kettani

**Affiliations:** 1 Anesthesia and Critical Care, Cheikh Khalifa International University Hospital, Mohammed VI University of Health Sciences, Casablanca, MAR

**Keywords:** covid-19, covid-19 immunology, gender disparities, gender-based differences, sex differences

## Abstract

Background

Severe acute respiratory syndrome coronavirus 2 (SARS-CoV-2) was identified as the cause of an outbreak of severe acute respiratory syndrome in Wuhan City, China in December 2019. Since then, it has caused a worldwide pandemic with over six million deaths. Many studies have been published on coronavirus disease 2019 (COVID-19), but only a few have compared the outcomes of COVID-19 between males and females, especially in African countries and the Middle East. Studies published to date on this topic report that male patients infected with SARS-CoV-2 have worse outcomes than females. This study aimed to compare sex differences in COVID-19 outcomes in a tertiary care hospital in Casablanca (Morocco).

Methodology

We included all adult patients hospitalized for COVID-19 infection in Cheikh Khalifa Hospital. We categorized patients according to gender and analyzed the characteristics and outcomes of each group.

Results

In total, 134 patients with polymerase chain reaction (PCR)-confirmed COVID-19 were admitted during the study period. These included 72 (53.7%) men and 62 (46.4%) women. The median age of the patients was 53 years (interquartile range, IQR = 36-64). Men were significantly older than women (58 vs. 44) but there was no significant difference in comorbidities on comparing men and women. Values of median C-reactive protein (35 vs. 4), ferritin (326 vs. 72), and lactate dehydrogenase (264 vs. 208) were significantly higher in men. Lymphopenia was significantly more important in men (1.2 vs. 1.7), and the ratio of neutrophil/lymphocytes was significantly higher in men (3.37 vs. 1.84). Men had greater disease severity, with significantly higher intensive care unit admission (48.6% vs. 16.1%) and higher hospital mortality (18.1% vs. 1.6%).

Conclusions

COVID-19 gender disparities may be due to sex differences in the inflammatory response. These can be explained by the role of sex hormones and sex chromosomes on immune cells and their regulatory genes.

## Introduction

Since severe acute respiratory syndrome coronavirus 2 (SARS-CoV-2) emerged in China at the end of 2019, it has caused a global pandemic, with more than 500 million reported infections and six million deaths. Early literature from China suggested a gender difference in both the prevalence and mortality rate of coronavirus disease 2019 (COVID-19) [1,2].

As COVID-19 spread, an impressive amount of data from countries around the world was published. Many of these concluded that the prevalence of COVID-19 was similar in both sexes, but that the mortality rate was higher in men [3,4]. Other studies have shown that susceptibility to SARS-CoV-2 infection and its prevalence differs between the sexes, as does mortality [5-7].

However, few studies on COVID-19 have compared outcomes between men and women, especially in African countries and the Middle East. Indeed, African countries still do not provide statistics on gender differences in COVID-19. Understanding the reasons for these gender disparities is fundamental to developing targeted strategies for managing this pandemic.

Casablanca, the biggest city in Morocco, is located in North Africa. The Cheikh Khalifa International University Hospital (CKIUH) is a tertiary care hospital that has managed a large number of COVID-19 patients. The objective of our study was to describe and analyze sex disparities in patients hospitalized for COVID-19 infection in the CKIUH.

## Materials and methods

Study design

This is a retrospective, observational, single-center study of patients admitted to CKIUH for COVID-19 between March 18, 2020, and May 20, 2020. This study compares demographic data, clinical characteristics, biological parameters, and evolution between male and female patients infected with SARS-CoV-2.

Eligibility criteria

All adult patients (over 18 years old) hospitalized for confirmed COVID-19 infection during the study period were included. The diagnosis of COVID-19 was based on a positive result on the reverse transcriptase-polymerase chain reaction (RT-PCR) assay collected on a nasopharyngeal swab. We categorized patients into two groups according to gender.

Data collection

We used our institutional software (DxCare) to collect electronic medical record data.

Statistical analysis

To describe the male and female groups, we used medians for continuous variables and frequencies for categorical variables. To compare the difference between the two populations, we used Student’s t-test or Mann-Whitney U test for continuous variables. For categorical variables, we used the chi-square test or Fisher’s exact test.

Ethical considerations

The use of patient data was approved by the institutional scientific and ethical committees of CKIUH and Mohammed VI University of Health Sciences (UM6SS) (approval number: CE_UM6SS/1/06/2020 - April 3, 2020). Data collection was carried out with respect to patient information confidentiality.

## Results

In total, 134 patients with PCR-confirmed COVID-19 were admitted during the study period. There were 72 (53.7%) men and 62 (46.4%) women. The median age of the patients was 53 years (interquartile range, IQR = 36-64). Men were significantly older than women (58 vs. 44 years). There was no significant difference in the comorbidities studied (hypertension, diabetes, cardiovascular disease, and asthma) when comparing male and female patients. Clinically, a greater proportion of men in our series suffered from asthenia during their hospitalization (Table 1).

**Table 1:** Demographic and clinical characteristics of patients. a: expressed in median (quartiles); b: expressed in frequency (%)

Characteristics	Total (n = 134)	Male (n = 72)	Female (n = 62)	P-value
Age (years)^a^	53 (36–64)	58 (41–65)	44 (30–61)	0.008
Comorbidities^b^				
Diabetes	19 (14.2%)	21 (29.2%.)	15 (24.2%)	0.460
Hypertension	36 (26.9%)	12 (16.7%)	7 (11.3%)	0.562
Cardiac disease	16 (11.9%)	10 (13.9%)	6 (9.7%)	0.595
Asthma	10 (7.7%)	5 (7.4%)	5 (8.1%)	1
Signs and symptoms^b^				
Asthenia	24 (17.9%)	21 (29.2%)	3 (4.8%)	<0.001
Fever	60 (44.8%)	38 (52.8%)	22 (35.5%)	0.056
Headache	20 (14.9%)	9 (12.5%)	11 (17.7%)	0.469
Myalgia	29 (21.6%)	20 (27.8%)	9 (14.5%)	0.092
Agueusia	26 (19.4%)	11 (15.3%)	15 (24.2%)	0.273
Anosmia	24 (17.9%)	10 (13.9%)	14 (22.6%)	0.259
Dry cough	61 (45.5%)	37 (51.4%)	24 (22.6%)	0.166
Dyspnea	39 (29.9%)	25 (34.7%)	14 (15.7%)	0.132
Abdominal pain	19 (14.2%)	12 (16.7%)	7 (11.3%)	0.460
Diarrhea	29 (21.6%)	15 (20.8%)	14 (22.6%)	0.836
Vomiting	23 (17.2%)	12 (16.7%)	11 (17.7%)	1

Overall, the proportion of laboratory abnormalities was significantly higher in men hospitalized with COVID-19. This was mainly manifested by lymphopenia (1.2 vs. 1.7) and an elevated neutrophil/lymphocyte ratio (3.37 vs. 1.84), two markers of COVID-19 severity. Inflammatory biomarkers such as C-reactive protein (CRP) (35 vs. 4), lactate dehydrogenase (LDH) (264 vs. 208), and ferritin (326 vs. 72) were significantly higher among men. However, D-dimer did not differ significantly between the two groups. For radiological parameters, COVID-19 reporting and data system (CO-RADS) was significantly higher in men than in women (33.3% vs. 11.3%). The biological and radiological data are presented in Table 2.

**Table 2:** Radiological and laboratory findings on admission. a: expressed in frequency (%); b: expressed in median (quartiles) CO-RADS: COVID-19 reporting and data system; LDH: lactate dehydrogenase; CRP: C-reactive protein; N/L: neutrophils/lymphocytes

Radiological and laboratory findings	Total (n = 134)	Male (n = 72)	Female (n = 62)	P value
Chest scan CO-RADS^a^	31 (23.1%)	24 (33.3%)	7 (11.3%)	0.004
Hemoglobin^b^ (g/dL)	14.0 (12.8–15.0)	14.6 (14.0–15.6)	13.1 (12.2–13.7)	0.0001
White blood cell^b^ (×10^9^/L)	6.34 (4.72–7.40)	6.8 (5.2–8.3)	5.6 (4.4–7.0)	0.007
Neutrophils^b^ (×10^9^/L)	4.03 (2.68–5.45)	4.7 (3.3–6.3)	3.3 (2.2–4.6)	0.0001
Lymphocytes^b^ (×10^9^/L)	1.45 (0.98–2.02)	1.2 (0.8–1.7)	1.7 (1.2–2.1)	0.001
N/L	2,60 (1.68–4.61)	3.37 (2.21–5.8)	1.84 (1.16–3.43)	0.0001
Platelets^b^ (×10^9^/L)	266 (225–339)	222 (163–293)	224 (200–286)	0.321
CRP^b^ (mg/L)	11.8 (2.8–71.2)	35 (7–111)	4 (2–25)	0.0001
Procalcitonin^b^ (ng/mL)	0.05 (0.05–0.31)	0.05 (0.05–0.16)	0.05 (0.05–0.05)	0.233
LDH^b^ (U/L)	228 (178–281)	264 (205–338)	208 (164–234)	0.001
Ferritin^b^ (µg/L)	174 (68–464)	326 (213–925)	72 (36–164)	0.0001
D-dimer^b^ (µg/L)	0.54 (0.30–0.87)	589 (271–936)	450 (333–736)	0.544

In total, 45 (33.6%) patients were admitted to the intensive care unit (ICU), with hospital mortality of 10.4%. Men had greater disease severity, with significantly higher ICU admission (48.6% vs. 16.1%) and higher hospital mortality (18.1% vs. 1.6%) (Table 3).

**Table 3:** Complications during hospitalization. a: expressed in frequency (%) ICU: intensive care unit

	Total (n = 134)	Male (n = 72)	Female (n = 62)	P-value
Severe form (ICU)^a^	45 (33.6%)	35 (48.6%)	10 (16.1%)	0.0001
Hospital mortality^a^	14 (10.4%)	13 (18.1%)	1 (1.6%)	0.002

## Discussion

We provide data from a North African country during the first wave of COVID-19 infection. Data from an African area is missing in international studies, especially regarding gender disparities. Initial reports from China, the United States, and Europe provided preliminary evidence of severe COVID-19 outcomes among men [2,7,8]. However, recent studies suggest that females may be at higher risk for COVID-19 [9,10] while other studies show no gender differences in SARS-CoV-2 infections [11]. Some studies have suggested that COVID-19 gender disparities may reflect demographic factors, health profiles, and social and cultural differences between different countries [12]. We showed in this study that the inflammatory response to COVID-19 (CRP, LDH, and ferritin levels) was significantly higher in men. This can explain the increased severity of the disease and mortality observed in men.

It has been shown that comorbidities are important predictors of severity in COVID-19 patients [4]. However, in this study, there was no significant difference between the two groups. This work concludes that men in Morocco may be at higher risk for severe forms of COVID-19, although it is a descriptive study and inferential statistics were not used. In the literature, we found many mechanisms that may explain this observation.

In humans, several genes contained in the sex chromosomes X and Y play roles in immune responses. They are present on the X chromosome such as pattern recognition receptors (PRRs), toll-like receptors (TLRs), costimulatory molecules, and transcription factors [13,14]. On the X chromosome in women, the biallelic higher expression of immunomodulatory genes may explain their stronger response to viral infection including COVID-19 [14,15]. On the other hand, some Y-linked genes that have an overexpression in men could be responsible for the higher viral load and hyperinflammatory response, especially *sex-determining region Y* (*SRY*) and *SRY-box transcription factor 9* (*SOX9*) [16].

The angiotensin-converting enzyme 2 (ACE-2) receptor has been described as the receptor for SARS-CoV-2 entry into alveolar type 2 pneumocytes [17]. Despite this negative role, the ACE-2 receptor also has a positive role in organic defense by inactivating the active forms of angiotensin I and II and reducing the chances of pulmonary edema during COVID-19 infection [18]. The ACE-2 receptor gene is located on the X chromosome (Xp22.2) and is, therefore, characterized by a higher expression in women [19]. This may explain why men are less protected than women.

Another mechanism of SARS-CoV-2 entry is the transmembrane serine protease 2 (TMPRSS2) which is required for priming the SARS-CoV-2 viral protein S. This protein is important for the pathogenesis of COVID-19 [17]. Data have also shown an association between male sex and increased expression of TMPRSS2 [20] through androgen receptors (ARs) [21]. Men have a higher expression of ARs than females due to dihydrotestosterone (DHT) [22]. This suggests that high levels of testosterone can lead to an important number of COVID-19 cases and an increased mortality rate in men. However, an Italian cohort study of 31 COVID-19 patients demonstrated that in men lower testosterone levels could increase the risk of serious infection [22].

The effect of sex hormones on innate and adaptive immunity can also explain the sex-related differences in COVID-19. In the literature, females are associated with more robust cell-mediated and humoral immune responses [23]. We know that estrogens could regulate many immune cells such as macrophages, B cells, T cells, and natural killers by acting on their estrogen receptors [24]. It has been further demonstrated that estrogens attenuate the production of pro-inflammatory cytokines interleukin (IL)-12 and IL-6 [25]. This could indicate a possible role of estrogens in preventing cytokine storm syndrome associated with COVID-19. Studies have also shown that estrogens promote the formation of immunoglobulin G (IgG) and immunoglobulin M (IgM), while testosterone inhibits their synthesis [26]. A recent study in patients with COVID-19 showed higher levels of IgG and IgM after COVID-19 infection, which indicates the importance of humoral immunity on COVID-19 outcomes [27]. Both of these studies highlight estrogen’s potential to improve humoral immunity.

Other factors described in the literature could also contribute to the apparent gender differences in mortality. Smoking prevalence in men has been suspected to be a predictive factor of disease severity in COVID-19 [28]. Whereas women are known to have better hygiene practices and higher use of healthcare services than men [29]. This can predispose men to bad clinical outcomes.

Our study has some limitations. Data analysis was done retrospectively and concerned only one healthcare system. There was only one racial group, and the age of the two groups was different. Moreover, we did not adjust for any covariates, such as age, comorbidity, and health behaviors. Additionally, socioeconomic covariates such as socioeconomic status and education levels were not included in the analysis. Due to these limitations, our data are not representative of a generalized population. Other studies are necessary to determine whether sex differences persist across other ethnic groups.

## Conclusions

Most studies indicate that a gender-based disparity exists in COVID-19 clinical outcomes. Men are more associated with poor clinical outcomes, while women are reported to have a protective advantage against COVID-19 with fewer inflammatory reactions and lower viral loads. Our study also supports this statement and gives an overview of the North African COVID-19 outcome gender disparity. Several factors may explain these differences. This includes the gender differences in innate and adaptive immunity, the role of sex hormones, and the differences in gender-specific behavior. However, further studies and clinical trials based on gender immunological divergence in response to viral infection are still needed to confirm this theory.
